# Microbiome structure in healthy and pregnant women and importance of vaginal dysbiosis in spontaneous abortion

**DOI:** 10.3389/fcimb.2024.1401610

**Published:** 2025-02-17

**Authors:** Majid Eslami, Ramtin Naderian, Ariyan Ahmadpour, Ali Shushtari, Sahar Maleki, Parham Mohammadian, Arvin Amiri, Maryam Janbazi, Mohammad Memarian, Bahman Yousefi

**Affiliations:** ^1^ Department of Bacteriology and Virology, Semnan University of Medical Sciences, Semnan, Iran; ^2^ Health Policy Research Center, Institute of Health, Shiraz University of Medical Sciences, Shiraz, Iran; ^3^ Student Research Committee, Semnan University of Medical Sciences, Semnan, Iran; ^4^ Faculty of Medicine, Mazandaran University of Medical Sciences, Sari, Iran; ^5^ Department of Veterinary Medicine, Faculty of Veterinary Medicine, Babol Branch Islamic Azad University, Babol, Iran; ^6^ Department of Internal Medicine, School of Medicine, Semnan University of Medical Sciences, Semnan, Iran; ^7^ Department of Immunology, Semnan University of Medical Sciences, Semnan, Iran

**Keywords:** spontaneous abortion, dysbiosis, gynecological, pregnancy, vaginal microbiome

## Abstract

The vaginal microbiome of healthy women is dominated by *Lactobacillus* spp. A variety of illnesses, such as vaginosis, sexually transmitted infections (STIs), failed implantation, premature birth (PTB), and preterm pre-labor membrane rupture, are brought on by an unbalanced microbiota. Pregnancy is associated with a decrease in the metabolic capacity of the vaginal resident microbiome, which is consistent with a change to a less complex *Lactobacillus*-dominated microbiome. Age, race, sexual intercourse, smoking, IUD, contraception, lifestyle, and diet all affect the makeup of the vaginal microbiome. Moreover, physiological events including menarche, the menstrual cycle, pregnancy, menopause, and other hormonal changes have an impact on the vaginal microbiome. The vaginal microbiome is significantly disrupted by the menstrual cycle, with significant changes toward a more varied microbiota occurring around menstruation. Several major factors maintain or disrupt the vaginal microbiome including ethnic group, menstruation cycle, and pregnancy which are discussed in this section. In the index pregnancy, the vaginal microbiota of women who had already given birth, or had just experienced an induced or spontaneous abortion, was qualitatively and quantitatively different from that of women who were having their first child. Early pregnancy vaginal microbiome depletion is a risk factor for early pregnancy miscarriage. Although, early pregnancy miscarriage is not always caused by a high bacterial diversity and quantity of *lactobacilli*. *Lactobacillus* protects against pathogens through the production of antibacterial compounds such as lactic acid and bacteriocins.

## 1 Importance of vaginal microbiome in spontaneous abortion

The term “abortion” refers to the removal or evacuation of an embryo or fetus in order to end a pregnancy. Abortion is performed on women for a variety of causes around the world. An abortion can happen naturally, in which case it is sometimes referred to as a miscarriage, or it can be intentionally caused, in which case it is frequently referred to as an induced abortion. Induced abortion is a straightforward and frequent medical practice (Alibrandi et al., 2022). Every year, 121 million pregnancies—or over half of all pregnancies—are unplanned; 6 out of 10 unwanted pregnancies and 3 out of 10 pregnancies overall result in induced abortions. The most frequent consequence is recurrent spontaneous abortion (RSA), which affects 30 to 40 percent of pregnancies before 20 weeks. Miscarriages that happen on their own and can be brought on by a number of things, such as illness, trauma, genetic defects, or a mother’s and fetus’s incompatibility with one another’s biochemistry. More than 50% of miscarriage cases are still unknown even after these causes have been ruled out. According to current research, the makeup and ratios of the microbiota in the female genital tract have a significant impact on fertility and are linked to missed abortions due to vaginal microbiology and disordered vaginal microbiota (Al-Nasiry et al., 2020; Schreck et al., 2022).

In healthy women, the vaginal microbiome is dominated by *Lactobacillus* spp. which makes the environment efficient. The imbalanced microbiome causes various diseases, including vaginosis, sexually transmitted infections (STI), implantation failure, preterm birth (PTB), and preterm pre-labor rupture of the membranes (Zhao et al., 2021). Although the underlying mechanisms are not fully understood, there is evidence that the vaginal microbiome is connected to a woman’s history of miscarriage. The makeup of the vaginal microbiota has been linked to a higher risk of miscarriage (Zhao et al., 2021; Grewal et al., 2022; Guang et al., 2022).

The similarity in the microbiome of women who have not experienced miscarriage is higher than in women with a history of miscarriage (Zhang et al., 2022). Evidence suggests that 77.3% of miscarriages belong to the community-state types of microbiome not dominated by *Lactobacillus* (X. Liu et al., 2021). The host immune system is impacted by the gut and vaginal microbiota, which also causes an imbalance in cytokine levels (Y. Liu et al., 2021). The pathophysiology of unexpected pregnancy loss has been hypothesized to entail a dysregulation of cytokine networks. Yet, it is still unclear how gut microbial dysbiosis affects cellular immunological activity in miscarriage. Vaginal microbiota imbalances can pass through the uterus and generate chemokines that set off a local immunological reaction. Here, we find that serum levels of patients who have miscarried had significantly higher levels of IL-2, IL-17A, IL-17F, TNF, and IFN. According to correlation analysis, various microbe-associated metabolites are favorably connected to changes in Th1/Th17 cytokine levels, which are conditions linked to recurrent miscarriages (Eslami et al., 2019b; M Eslami et al., 2020a; Y. Liu et al., 2021).

## 2 Gynecological system microbiome: vaginal/endometrial/placenta microbiome

Five community types (CSTs) are used to categorize the vaginal microbiome: CST -I (dominated by *L. crispatus*), CST-II (dominated by *L. gasseri*), CST-III (dominated by *L. iners*), CST -V (dominated by *L. jensenii*), and CST-IV (dominated by anaerobic bacteria, including *Aerococcus, Atopobium, Prevotella, Dialister, Megasphaera, Gardnerella*, and *Sneathia*), with a small number of women falling into the latter group. *Lactobacillus* is the most microorganism found in the microbiome of a healthy vagina. The bacterium produces lactic acid and bacteriocins to keep the PH acidic, preventing pathogenic bacteria in the vagina. This process creates an acidic environment with a pH of around 3 (Koumentakou and Massouras, 1985; Nasioudis et al., 2017). This acidic environment suppresses the growth of most bacteria and other non-bacterial microbial agents such as *Candida* spp. and thus maintains a healthy vaginal environment. As a result, a *Lactobacillus*-dominated vagina is protected from infection and reduces the likelihood of miscarriage (Zhang et al., 2019; X. Liu et al., 2021; Günther et al., 2022). *Lactobacillus* spp. including *L. crispatus, L. iners, L. gasseri*, and *L. jensenii* can be influenced by two types of factors: physiological and exogenous. Physiological factors such as the menstrual cycle, hormonal changes, menopause, and pregnancy can alter the vaginal microbiota. Moreover, the combination of microbiota can be affected by some exogenous reasons such as stress, sexual habits, drugs, smoking, and diet (Eslami et al., 2021; Zhao et al., 2021).

The predominant bacteria in a healthy vaginal microbiome are *Lactobacillus* species. By producing lactic acid, they keep the pH of the environment slightly acidic, which inhibits the growth of dangerous germs. The sex hormone estrogen is important in maintaining this environment. The higher estrogen levels, which are generally seen during the reproductive years, encourage *Lactobacillus* development. Glycogen serves as a source of nutrients for *Lactobacillus* and is produced when estrogen promotes the maturation of vaginal epithelial cells. Indirectly supporting the formation of *Lactobacillus*, estrogen promotes a healthy vaginal microbiome (Shen et al., 2022).

A disturbance in the hormonal equilibrium, namely a reduction in estrogen levels, may have an adverse impact on the stability of the vaginal microbiota. This may happen while: (A) Menstrual Cycle: The menstrual cycle causes fluctuations in estrogen levels. Estrogen levels drop during the luteal phase (after ovulation), which may cause a brief reduction in *Lactobacillus* dominance. (B) Pregnancy: Progesterone levels rise dramatically while estrogen levels rise somewhat throughout pregnancy. Variations in the composition of the vaginal microbiome may be caused by this shift in hormones. (C) Menopause: There is a sharp drop in estrogen levels following menopause. This major hormonal shift may raise the risk of recurrent infections by causing a prolonged decrease in *Lactobacillus* populations and an increase in vaginal pH and (D) Medical Conditions: A number of illnesses, including polycystic ovarian syndrome (PCOS), have the potential to alter the vaginal microbiota and throw off the hormonal balance. A disturbance of the sensitive microbial equilibrium may have an impact on the implantation procedure or the development of the embryo’s nutrition. Dysbiosis-induced inflammatory reactions may damage the uterine lining or have a deleterious effect on the growing fetus (Saraf et al., 2021; Shen et al., 2022).

For several years, scientists hypothesized that the endometrium was a sterile and bacteria-free organ. Compared with the vaginal microbiota, the endometrial microbiota is a low biomass environment with an estimated bacterial load 100-10,000 times lower (Eslami et al., 2020b; Moreno et al., 2020; Elnashar, 2021). In contrast to the vagina, the number of *lactobacilli* decreases, while *Acinetobacter* and *Pseudomonas* increase. It should be mentioned that the probability of miscarriage is lower in an endometrium dominated by *Lactobacillus* (Zhao et al., 2021). *Lactobacillus* iners is the most commonly detected and only microorganism in early successful pregnancy that plays an important role in defense and basal function. The composition of the endometrial microbiome is as follows: *Lactobacillus* spp. (30.6%), *Pseudomonas* (9.09%), *Acinetobacter* (9.07%), *Vagococcus* (7.29%), and *Sphingobium* (5%) (Elnashar, 2021).

The placental microbiome evaluated by DNA is identical to that of the nonpregnant oral cavity. Some oral microorganisms such as *Fusobacterium nucleatum* may facilitate blood passage during placentation because they are capable to attach to the vascular endothelium, facilitating the predominant microbes such as *Escherichia coli*. The placental microbiome has a small but metabolically active microbiome. The microbiome is composed of the phyla *Bacteroidetes*, *Proteobacteria*, *Tenericutes*, *Firmicutes*, and *Fusobacteria* (Peerayeh et al., 2013; Aagaard et al., 2014). Colonization of the placenta with *Ureaplasma parvum* may be associated with spontaneous abortion (Teixeira Oliveira et al., 2021). There are some limitations to the study of the placental microbiome. The collection of samples from the placenta is invasive during pregnancy, so it may be difficult to study the placental microbiome. The placental microbiota is not affected by vaginal Group B *Streptococcal* (GBS) colonization, maternal weight, or mode of delivery, which is also of interest to us (Aagaard et al., 2014) (**Table 1**).

**Table 1:** Human studies about the effects of the vaginal microbiome on abortion.

Year of Study	Country	Kind of study	Sample size	Changes of Microbiome	Abortion	Results	References
2009	Nigeria	Prospective	150 women with septic abortion	132 patients grew organisms while the samples from 18 patients had no growth	Septic abortion	Incidence of abortion-related complications was 11% and septic abortion was 5.1%	(Oriji et al., 2015)
2017	USA	Prospective	155 women	A prior pregnancy history affects the microbiota	16 cases of abortion	Development in early gestation may differ between primiparous and multiparous women	(Nasioudis et al., 2017)
2018	China	Case-control	10 patients with unexplained RM and 10 healthy volunteers	In RM group, ↑*Atopobium*, *Prevotella*, and *Streptococcus*, and in control group ↑*Lactobacillus* and *Gardnerella*	None of the women were pregnant	Dysbiosis in women with RM	(Zhang et al., 2019)
2019	USA	Case-cohort	273 women	No changes stability of the vaginal microbiota	spontaneous abortion (<20 weeks gestation)	*Lactobacillus* may be associated with modestly ↑fecundability.	(Lokken, 2019)
2020	China	Retrospective cohort	85 Patients	Lose of *Lactobacillus*	10 Spontaneous abortions (weeks 14 – 28)	An absence of *Lactobacillus* spp. are potential risk factors that predict subsequent cerclage failure	(Fang et al., 2020)
2020	Denmark	Cohort	A total of 730 (GW24) and 666 (GW36) vaginal samples from 738 unselected pregnant	Changes in vaginal microbiota from 24-36 weeks correlated with bacterial vaginosis	With a p-value of 0.305	Among women with vaginosis in week 24, 47% had vaginosis in week 36.	(Haahr et al., 2022)
2020	Netherlands	Prospective observational study	85 women	↓ Lactobacillus spp. ↑*Staphylococcus* spp.	No abortion	Urinary microbiome before IVF/IVF-ICSI treatment as a predictor for clinical pregnancy outcomes	(Koedooder et al., 2021)
2021	Brazil	Cross-sectional	89 women spontaneous Abortion (case) and 20 women NVD (control)	Was not considered and no investigation was performed on the presence of chronic endometritis.	Spontaneous Abortion	↓ immune response and activating the extrinsic apoptotic pathway, causing spontaneous abortion	(Teixeira Oliveira et al., 2021)
2021	Brazil	experimental	36 gilts (244 ± 22 days old)	Gilts in crates showed changes in microbiome	No abortion	the vaginal microbiota depends on the bacterial exposure	(Alves et al., 2022)
2021	China	cohort	50 women	No significant change	Group E (empty-sac miscarriage ) Group M (missed miscarriage) Group P (normal pregnant)	Patients with early pregnancy miscarriage had a significantly different vaginal microbiota profile	(Liu et al., 2021)
2022	China	Cohort	454 women	*↑L. crispatus, L. gasseri*, *L. iners, L. jensenii*, and *L. reuteri* ↓*Prevotella*, *Atopobium*, *Acinetobacter*, and *Sneathia* during pregnancy	–	*Lactobacillus* spp. were more prevalent during pregnancy in the PROM-PTB cases	(Zhang et al., 2022)
2022	Finland	Case-control	324 Finnish women between 37_42 weeks of gestation	↑ *L. crispatus*	49 (26.1%) of the studied women	18.5% were taken at elective CS, 29.6% at delivery between 37.1-41 and 51.8% at post-term visit at the maternal outpatient clinic	(Kervinen et al., 2022)
2022	UK	cohort	167 Women (93 miscarriages, 74 delivered)	↑ *Lactobacillus* spp. in women with abortion	Week 5-8 (n=35) Week 8-10 (n=84) 10-14 (n=48)	The vaginal microbiota plays an etiological role in euploid miscarriage	(Grewal et al., 2022)
2022	China	Case-control	87 women	↓gut microbial diversity, ↑ratio of *Firmicutes* to *Bacteroidetes*	Missed miscarriage (n= 63) Elective abortion (n=24)	↓Rectal microbiota diversity and a pro-inflammatory tendency in the miscarriage group.	(Guang et al., 2022)
2022	China	Case-control	120 patients	Bacterial richness and diversity remained unchanged when patients were treated with metformin.	All of the patients had an abortion	↑ abundance of vaginal *Lactobacillus* spp. in patients	(Zhao et al., 2021)
2022	Italy	Cohort	63 Caucasian women with a successful pregnancy and 9 women who had a first-trimester miscarriage	During pregnancy ↓ overall diversity, ↑stability. ↑ *Lactobacillus* and ↓ *Prevotella*, *Atopobium*, and *Sneathia*.	first trimester	↑ Prevotella in intrapartum antibiotic prophylaxis for GBS ↑*Fusobacterium* in women suffering the first trimester	(Severgnini et al., 2022)
2022	Egypt	Case-control	219 pregnant (167 Women with negative BV and 52 With positive BV)	no statistically significant relation between BV	38 women miscarried during the first trimester and 181 women miscarried after 13 weeks.	no statistically significant relation between BV	(Mansour et al., 2022)

↑ = increase, ↓= decrease.

## 3 Microbiome structure in pregnancy and postpartum period

The human vagina is an ecosystem with up to 10^9^CFU/mL vaginal fluid that includes a microbiome such as aerobic and anaerobic bacteria. It is discovered that a balanced microbiome is dominated by *Lactobacillus* spp. which protects the environment due to these ways: 1) Blocking the attachment of harmful microorganisms, when a biofilm forms on the epithelial cell receptors. 2) *Lactobacillus* spp. generate antimicrobial products like hydrogen peroxide (H_2_O_2_), lactic acid, and bacteriocins which inhibit growth. H_2_O_2_ is produced primarily from *L. crispatus* and *L. jensenii*. The pH of the vagina is between 3.5 and 4.5 on hydrolysis and lactic acid is produced by the fermentative catabolism of carbohydrates, mainly glucose, which releases glycogen. 3) Association with pathogens. These outcomes improve epithelial cells’ barrier function and promote host defenses (Vazquez et al., 2019).

Studies indicate that the makeup of the vaginal microbiota, specifically the quantity of *Lactobacillus* species present, may impact the course of a pregnancy, including the possibility of an early birth. It is well known that *Lactobacillus* species are essential for keeping the vagina’s pH acidic and for generating antimicrobial compounds that aid in preventing the growth of dangerous bacteria (Petrova et al., 2015). One important genus of probiotic bacteria that is important for vaginal health and balance is *Lactobacillus*. These helpful microbes stop vaginal infections by making the environment unfavorable for pathogen growth. A significant link has been seen between the risk of preterm delivery (PTD) and a decline in *Lactobacillus* species in the vaginal microbiota. This is how the link is broken down:

(A) *Lactobacillus* and Vaginal Health: In a healthy vaginal microbiota, *Lactobacillus* species predominate. By producing lactic acid, they keep the pH of the environment slightly acidic, which inhibits the growth of dangerous germs. (B) Disruption of Microbiota: The balance of the vaginal microbiome is upset by a reduction in *Lactobacillus*. *Gardnerella vaginalis* and other potentially harmful bacteria may proliferate as a result of this. (C) Inflammation and Infection: An infection that may rise from the vagina to the uterus can be brought on by the expansion of these microorganisms and (D) Preterm Labor: It is believed that infections and inflammations of the reproductive system may play a role in the onset of preterm labor. According to studies, certain *Lactobacillus* species like *L. jensenii*, *L. crispatus*, and *L. gasseri* are better than others at preserving vaginal health (Hočevar et al., 2019; Rosca et al., 2020; Argentini et al., 2022).

There are two patterns of the microbiome in pregnant women. The microbiome of one group remains *Lactobacillus*-dominated throughout pregnancy while the microbiome of the other fluctuates throughout pregnancy. While the microbiome of one group was previously more varied in the first trimester, it became *Lactobacillus*-dominated in the second trimester, but it returned to its original state after giving birth in the third trimester (Serrano et al., 2019).

During pregnancy, the vaginal microbiomes diversity declines while its stability rises. Furthermore, during pregnancy, there is a considerable rise in the amount of the dominating *Lactobacillus* in the vagina, which lowers the pH and boosts the vagina’s resistance to pathogenic microbes. Pregnancy is related to a reduction in the resident vaginal microbiomes metabolic capability, which is consistent with a shift towards a less complicated *Lactobacillus*-dominated microbiome (Nasioudis et al., 2017).

During pregnancy, *L. crispatus* was associated with less variation and more consistency (Zhao et al., 2021). The *Lactobacillus* can protect the vagina and *L. Crispatus* is essential for maintaining the integrity of the vaginal environment in pregnant women. Conversely, excessive estrogen levels can cause *lactobacilli* to break down lactic acid and glycogen. Moreover, the low pH in the vagina is also helpful for *lactobacillus* development and bacterial prevention. Consequently, vaginal pH can serve as a predictor of vaginal infection (Guang et al., 2022).

A study reported that *lactobacillus*-dominant microbiomes were less frequent in African-American women in the first trimesters, while European American women had more *Lactobacillus* vagitypes during pregnancy (Bayar et al., 2020). The *L. iners* vagitype was much more prevalent in women of African descent, while *G. vaginalis* and other vagitypes often linked to dysbiotic conditions were less prevalent. There were no observable changes in the vagitypes of non-African women whose early pregnancies were above 75% dominated by *Lactobacillus* vagitypes. In the second trimester, pregnant women of African descent’s vaginal microbiomes show a higher frequency of Lactobacillus species, due to an increase in the predominance of the *L. iners* vagitype (Nasioudis et al., 2017).

Pregnancy-related vaginal microbiota diversity is lower than that in the postpartum period (Günther et al., 2022). The vaginal microbiota is significantly different during pregnancy than after delivery (Zhang et al., 2022). During the postpartum period, it was discovered that the vaginal microbiome was more varied. The proportions of *Prevotella bivia* and *Streptococcus anginosus* were much higher, while *Lactobacillus* was significantly less common. *Lactobacillus* spp. dominated vaginal communities during the first and third trimesters, whereas postpartum populations showed lower numbers of *lactobacilli* and were more diverse (Koumentakou and Massouras, 1985).

First and third-trimester vaginal communities are often very similar. Approximately *Lactobacillus* spp. vaginal communities dominated 79% of the first trimester samples and 78% of the third-trimester samples. Conversely, the combination of the postpartum vaginal groups was significantly more diverse. Low levels of *Lactobacillus* spp. and a variety of bacteria including *G. vaginalis*, *Prevotella* spp., *Streptococcus* spp., and many others, were present in approximately 77% of vaginal communities after birth (Nunn et al., 2021). *L. crispatus, L. Jensen*, and *L. gasseri* were much less common in postpartum vaginal communities, whereas *L. iners* was not. Meanwhile, vaginal microbiota collected during the postpartum period contained significantly more *Prevotella bivia* and *Streptococcus anginosus*. Some postpartum specimens had increased abundances of *Streptococcus agalactiae*, but generally, these variations were not significant statistically (Koumentakou and Massouras, 1985; Eslami et al., 2019a).

**Figure 1:**
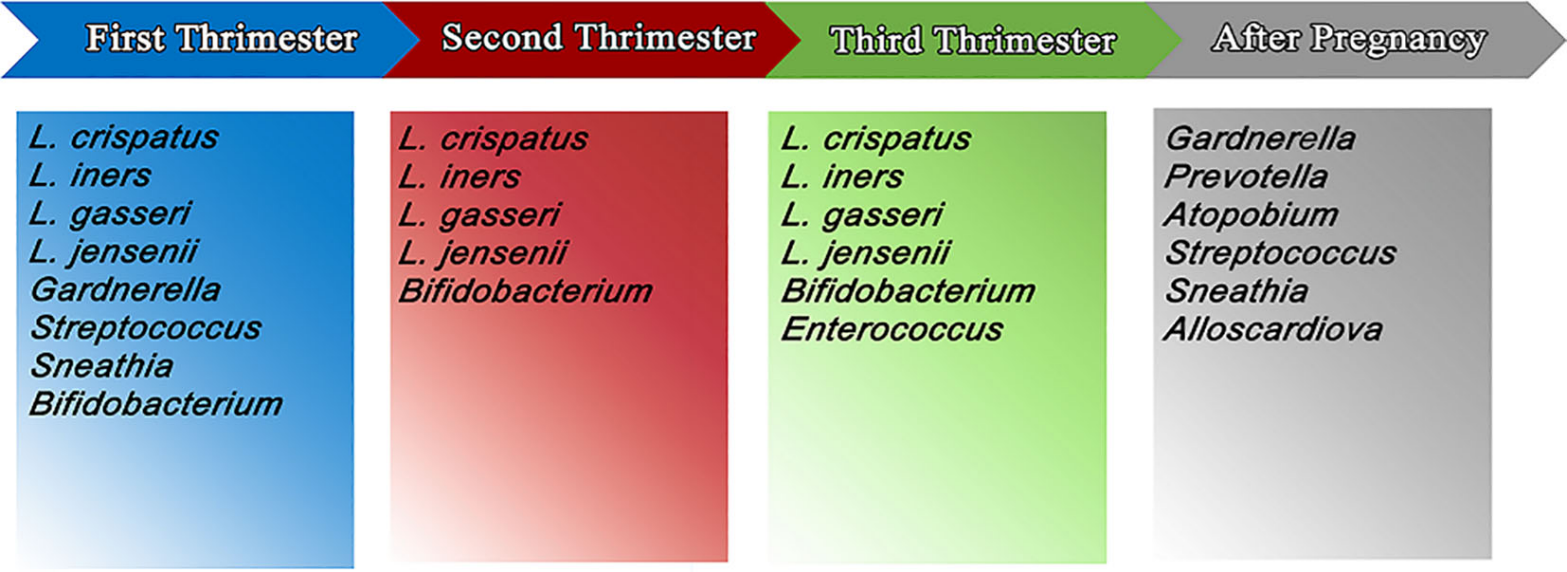
The most important and the most abundant microbiomes in vagina in first, second and third trimester of pregnancy and postpartum.

There is evidence that the *Lactobacillus*-deficient postpartum microbiota profile can persist up to a year after birth. Finally, the vaginal microbiota in the late third trimester differed according to how long the pregnancy had been going on. It was discovered that nulliparous and multiparous women had considerably different vaginal microbiota, demonstrating that the microbiome right prior to delivery reflects the reproductive history (Salek Farrokhi et al., 2020; Kervinen et al., 2022) (**Figure 1**, **Table 1**).

## 4 Causes of vaginal microbial change

The vaginal microbiome is a dynamic microbial environment that interacts with the environment. Age, race, sexual intercourse, exogenous toxins such as smoking, contraceptive use, IUD, lifestyle, and diet are factors that contribute to the composition of the vaginal microbiome. Moreover, physiological factors including menarche, the menstrual cycle, pregnancy, menopause, and other hormonal changes have an impact on the vaginal microbiome (Ghasemian et al., 2018; Zhao et al., 2021).

Because the biochemical pathways leading to glycogen production and release depend on sex hormones, estrogen, in particular, this means that the vaginal microbiome exhibits a great diversity during life periods including childhood (pre-menarche) and postmenopausal period with low plasma levels of sex hormones of microbiota with less abundant *Lactobacillus* spp. and more anaerobic gram-positive cocci and gram-negative rods (García-Velasco et al., 2017). According to a number of studies, low levels of estradiol during menstruation cause low glycogen deposition and, as a result, a lesser presence of Lactobacillus, which leads to a higher pH that promotes the growth of anaerobic BV bacteria. The vaginal epithelium of White and Asian women is primarily colonized by types 1, 2, 3, and 5 (*lactobacilli*-dominated microbiome), while type 4 is mainly observed in Hispanic and Black females with a prevalence of 34/3% and 38/9%, respectively. A less acidic vaginal pH and a more diverse vaginal microbiota in this CST type reflect the reduction in lactate-producing *Lactobacillus* spp. Population and dominance of the vaginal microbiome by anaerobic bacteria, particularly *Gardnerella* spp (Günther et al., 2022).

The vaginal microbiome is significantly disrupted by the menstrual cycle, with significant changes toward a more varied microbiota occurring around menstruation. These changes are mostly noted in women with natural cycles in contrast to oral contraceptive and levonorgestrel intrauterine device (LNG-IUD) users and the reason for these changes is generally considered to be related to a decrease in the estradiol level in the menstruation phase in contrast to the follicular and luteal phase. Some other factors such as the protective effect of the local release of LNG-IUD hormones on the vaginal epithelium and blood flowing through the vagina in the menstruation phase, thus providing an iron-riched environment, have an unknown significance (Krog et al., 2022).

A previous experiment also discovered a clear connection between oestradiol levels and *Lactobacillus* species generally and *L. crispatus* specifically (Krog et al., 2022). In the meta-analysis of shotgun metagenomics datasets of 1312 vaginal samples of pregnant (n=333) and non-pregnant (n=979), healthy women (Milani et al., 2018). Found that Pregnancy reduces the biodiversity of the vaginal microbiota, thus reducing the risk of BV and its adverse effects on pregnancy outcome. Genomic and metabolic analysis of CST I revealed that this CST type produces bacteriocins (mostly type 3 bacteriocins) about 10 times more than other CST types and encodes metabolic pathways which help in the catabolism of toxic metabolites secreted from the vaginal epithelium (Li et al., 2020). These characteristics indicate the ability of CST I *lactobacillus* spp. (the CST which is generally considered to maintain a healthy vaginal microbiome) to dominate the vaginal microbiome and reduce the abundance of other species thus reducing the biodiversity of vaginal microbiota (Milani et al., 2018).

**Figure 2:**
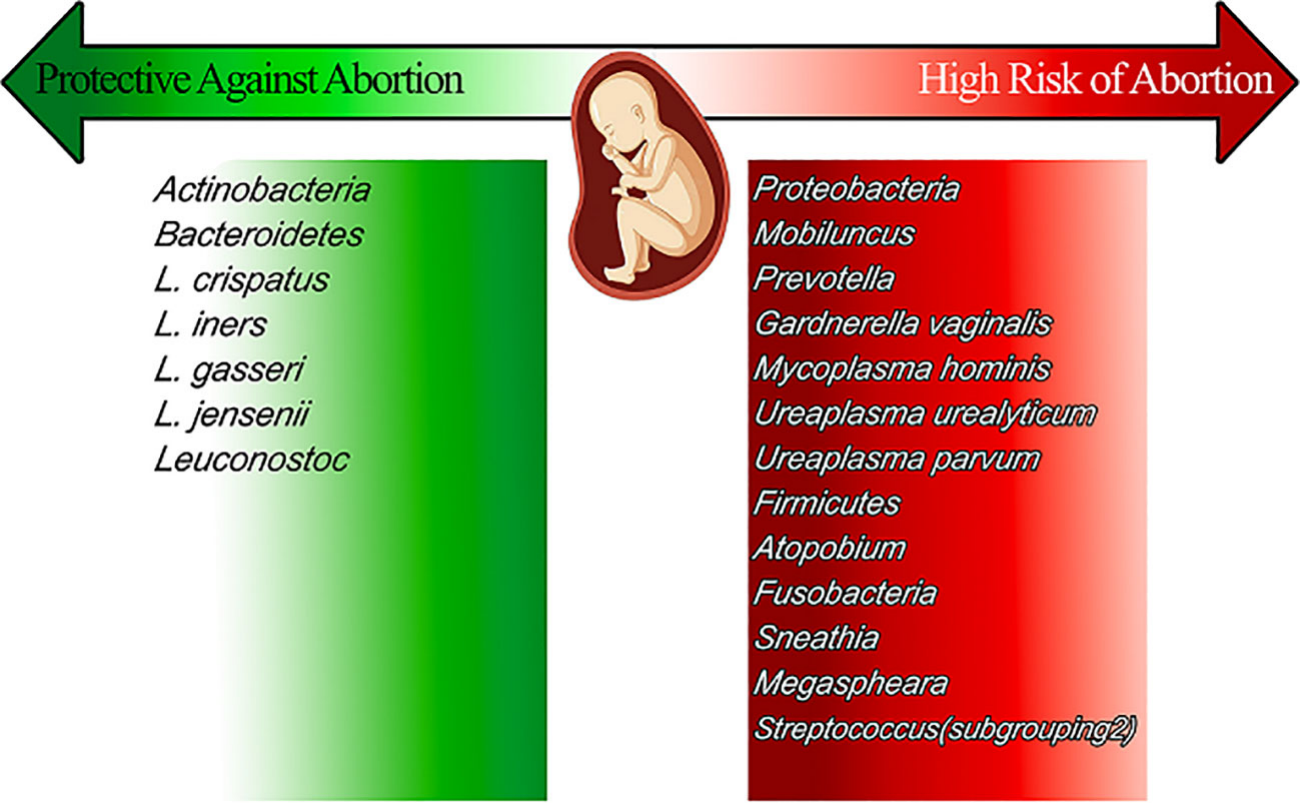
The vaginal microbiomes that present during pregnancy, reviews their effect on pregnancy and sort them into the high-risk group and protective ones.

In general, a *Lactobacillus* spp. dominant vaginal microbiota is considered the optimal vaginal microbiome where *lactobacilli* produce lactate through fermentation of a glycogen-enriched environment. This process creates an acidic environment with a pH of around 3 (Koumentakou and Massouras, 1985; Nasioudis et al., 2017). This acidic environment suppresses the growth of most bacteria and other non-bacterial microbial agents such as *Candida* spp. and thus maintains a healthy vaginal environment. Because the biochemical pathways leading to glycogen production and release depend on sex hormones, estrogen, in particular, this means that the vaginal microbiome exhibits a great diversity during life periods including childhood (pre-menarche) and postmenopausal period with low plasma levels of sex hormones creating a microbiota with less abundant Lactobacillus spp. and more anaerobic gram-positive cocci and gram-negative rods (García-Velasco et al., 2017). Several major factors maintain or disrupt the vaginal microbiome including ethnic group, menstruation cycle, and pregnancy which are discussed in this section. Some minor factors including age, sexual intercourse, hygiene practice, diet, and smoking are discussed separately (**Figure 2**).

### 4.1 Racial disparities in the vaginal microbiome

Several studies have found differences in the composition of the vaginal microbiome among different racial and ethnic groups. For example, African American women tend to have a higher abundance of bacteria from the genus *Lactobacillus*, which is associated with a healthy vaginal microbiome, compared to women of other ethnicities. However, they also have a higher prevalence of bacterial vaginosis (BV), a condition characterized by an overgrowth of harmful bacteria in the vagina. Although a wide range of bacteria can cause BV, some significant anaerobic species are frequently associated with the illness: *Bacteroides* spp. are known to cause BV, can overgrowth in the vagina, and are common inhabitants of the gut microbiome. Another typical gut flora that can grow out of control in the vaginal environment during BV is *Prevotella* spp., and *Peptostreptococcus* species that these anaerobic bacteria contribute to the distinctive symptoms and indicators of BV and are frequently associated with it. The growth of anaerobes in BV upsets the delicate vaginal ecosystem, which can have a range of detrimental implications. The pH rises in the vagina because anaerobic bacteria do not produce lactic acid. The pH change promotes the growth of additional anaerobes, further upsetting the ecosystem’s delicate equilibrium. Additionally, some anaerobic bacteria can form biofilms, which adhere to vaginal epithelial cells and function as a barrier to host defenses and medications. Anaerobic bacteria produce volatile compounds such as amines, which are the cause of the characteristically fishy odor associated with BV (Tomas et al., 2020; Chen et al., 2021).

On the other hand, Hispanic and Asian women tend to have a lower abundance of *Lactobacillus* and a higher abundance of other bacteria, such as *Gardnerella vaginalis*, which are associated with an increased risk of BV and other vaginal infections. These racial differences in the vaginal microbiome may be due to genetic and environmental factors, such as diet, sexual behavior, and hygiene practices. They can also have implications for women’s health, as an imbalance in the vaginal microbiome can lead to an increased risk of sexually transmitted infections, preterm birth, and other gynecological conditions. Therefore, understanding these differences and their impact on women’s health is important for developing targeted interventions and treatments (Ravel et al., 2011; Fettweis et al., 2014; Borgdorff et al., 2017).

To discuss racial differences in detail, we need to review the widely accepted CST classification method first proposed by Ravel et al. Healthy women’s vaginal microbiomes 396 healthy women of reproductive age with a range of ancestries were taken and examined using 16srRNA sequencing to identify different CST microbiome types, each with a particular dominating bacterial strain and metabolic profile. A total of five different types were observed. The vaginal epithelium of White and Asian women is primarily colonized by types 1, 2, 3, and 5 (*lactobacilli*-dominated microbiome), while type 4 is mainly observed in Hispanic and Black females with a prevalence of 34/3% and 38/9%, respectively. a less acidic vaginal pH and a more diverse vaginal microbiota in this CST type reflect the reduction in lactate-producing *Lactobacillus* spp. Population and dominance of the vaginal microbiome by anaerobic bacteria, particularly *Gardnerella* spp. (Ravel et al., 2011).

### 4.2 Menstrual cycle

Unknown causes include the blood moving through the vagina during the menstrual phase, creating an environment rich in iron, and the protective effect of the local release of LNG-IUD hormones on the vaginal epithelium (Krog et al., 2022). According to a number of studies, low levels of estradiol during menstruation cause poor glycogen deposition and, as a result, a lesser presence of Lactobacillus, which leads to a higher pH that promotes the growth of anaerobic BV bacteria. Moreover, recent research indicates a connection between oestradiol levels and Lactobacillus species generally and L. crispatus specifically (Krog et al., 2022).

### 4.3 Vaginal dysbiosis

Vaginal dysbiosis is characterized by a more diverse vaginal microbiota with a lower proportion of *Lactobacillus* spp. compared to the other anaerobic bacteria such as *Gardnerella vaginalis*, *Prevotella*, and *Sneathia* spp. resulting in lower lactate and H_2_O_2_ production and a less acidic environment. This leads to bacterial overgrowth, which ends in a vicious cycle of the less acidic vaginal epithelium and more bacterial overgrowth (García-Velasco et al., 2017; Nasioudis et al., 2017; Gholiof et al., 2022). The Amsel criteria and the Nugent score are two diagnostic standards for bacterial vaginosis (BV). A clinical evaluation known as the Amsel criterion is based on the presence of clue cells in a wet mount, vaginal discharge, a vaginal pH > 4.5, and an amine odor (when vaginal secretions are treated with potassium hydroxide solution). Three of the four criteria must be met in order to diagnose BV. The Nugent scoring system is a lab-based technique that evaluates the amount of *lactobacilli* and other bacterial morphotypes in a vaginal swab as well as the Gram stain. The Amsel criteria are a trustworthy and effective diagnostic option when laboratory equipment is not readily available, despite the fact that this approach is regarded as the gold standard in diagnostics. It’s interesting to note that the relationship between the level of vaginal dysbiosis and the Nugent score and vaginal pH is often positive (Gholiof et al., 2022).

The hallmark symptom of BV is a stinking vaginal discharge, although occasionally there are no obvious indications of infection or inflammation. The clinical diagnosis of BV is based on a dysbiosis of the vaginal microbiota. According to age, hygiene, race, education level, and economic condition, the prevalence of BV varies from 7% to 33% in women of reproductive age. STIs, including bacterial infections like *Chlamydia trachomatis* and *Neisseria gonorrhea* and viral infections like herpes simplex virus type 2 (HSV-2), human *papillomavirus* (HPV), and human immunodeficiency virus, are more common in those who have BV (HIV -1). BV is an independent risk factor for negative pregnancy outcomes, such as lower fertility, early and late miscarriages, premature rupture of membranes (PPROM), and preterm delivery, in addition to increasing STI acquisition (Lewis et al., 2017). It has been demonstrated that a number of variables, such as smoking, vaginal douching, menstruation, and new sexual partners, raise the risk of BV (Lewis et al., 2017; Noyes et al., 2018). Increased estrogen levels promote the stability of the vaginal microbiota and the growth of the lactobacilli population. Diet influences the vaginal microbiota via pro-inflammatory effects on the distal gut. Fat-rich foods increase the risk of vaginal dysbiosis, while calcium, vitamin E, and folic acid have a protective effect (Barrientos-Durán et al., 2020).

A novel and fascinating theory suggests that COVID-19 infection alters the vaginal microbiota in pregnant women, resulting in dysbiosis with decreased Lactobacillus abundance. An overview of studies confirming and refuting this theory is provided below: The following studies provide support for the hypothesis: “Alterations in vaginal microbiota among pregnant women with COVID-19”: Celik et al. conducted a significant study using a case-control approach to compare the vaginal microbiota of 19 pregnant COVID-19 women with 28 healthy controls. Along with an increase in *Bacteroidetes*, they observed a significant drop in *Firmicutes*, which includes *Lactobacillus*, and *Lactobacillus* in the COVID-19 group. This points to a change in the makeup of the bacteria that favors dysbiosis. The study also found that there may be a connection between vaginal dysbiosis and the severity of COVID-19, with women with moderate to severe symptoms showing greater levels of *Ureaplasma* (Celik et al., 2021).

The study “The perinatal microbiome and preterm birth” explores the shown advantage of a balanced vaginal microbiota in preventing premature birth, although it does not directly address this concept. It emphasizes how crucial *Lactobacillus* is to preserving an environment that is somewhat acidic and halting the spread of infections. This information offers a solid basis for comprehending how dysbiosis brought on by COVID-19 may raise the risk of premature birth. Research that could differ or need more research there isn’t much research out there now that directly challenges the hypothesis (Ailioaie and Litscher, 2021). Nevertheless, some research hasn’t found a direct connection with COVID-19 and vaginal dysbiosis in those who aren’t pregnant (Monsalve Rodríguez et al., 2023). Furthermore, for conclusive results, more study with larger cohorts is required.

**Figure 3:**
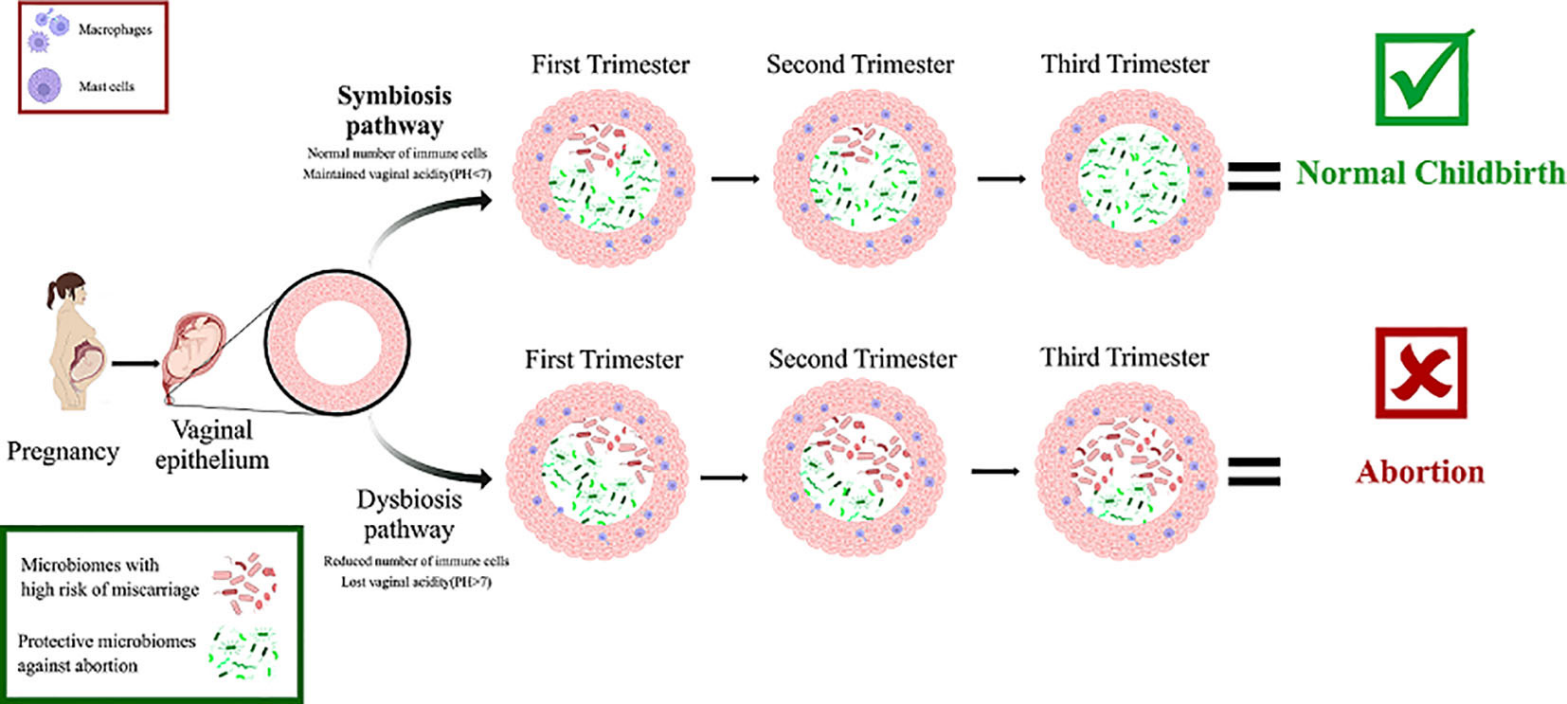
The symbiosis and dysbiosis pathways of vaginal microbiomes during pregnancy as you can see the microbiomes that participate in symbiosis protect the fetus from risk of abortion by keeping the pH level below 7, and keeping immune cells in normal amount. But in the other hand microbiomes that participate in dysbiosis increase the risk of abortion by increasing the pH level and reducing the number of immune cells.

BV and HPV are two sexually transmitted infections (STIs) that are linked. BV is a potential risk factor for HPV contamination through sexual intercourse, but it is unclear whether HPV infection is a risk factor for bacterial vaginosis or which of them precedes the other. Although it is clear that less dominant *Lactobacillus* spp. and a broader range of microbiota exist in individuals with HPV. The odds ratio for developing BV in HPV-infected patients is 2.35 in contrast to the odds ratio for acquiring HPV infection in BV patients of 1.83 (Lebeau et al., 2022). Smoking is one of the factors that cause vaginal dysbiosis and BV and can reduce the population of *Lactobacillus* in a dose-dependent manner. Gas and liquid chromatographic evaluation of the vaginal metabolome (670 vaginal metabolites) revealed significant differences in 12 metabolites including an increase in the concentration of nicotine, agmatine, cadaverine, putrescine, triptamine, tiramine, and reduced concentration of dipeptides in smokers (Nelson et al., 2018) Finally, vitamin D has been shown to have a protective effect against colonization of the vaginal microbiome by BV-associated bacteria by improving immunological function (Jefferson et al., 2019) (**Figure 3**).

## 5 Vaginal dysbiosis and spontaneous abortion

In miscarriages, the presence of *Lactobacillus* spp. is low, although the diversity and percentage of other *Lactobacillus* spp. were higher, including *L. aviaries, L. amylovorus, L. casei, L. delbrueckii, L. murinus, L. fructivorans, L. intestinalis, L. Plantarum, L. Reuters, L. similis, L. spicher*, and *L. zymase* (X. Liu et al., 2021). The likelihood of spontaneous abortion 5 times higher when the proliferation of *Gardnerella vaginalis* or *Mycoplasma* causes bacterial vaginosis. In addition, a severe maternal immune response caused by *Ureaplasma urealyticum*, *streptococcus*, *Neisseria gonorrhea*, and *Chlamydia trachomatis* can lead to spontaneous abortion (Mansour et al., 2022). *Lactobacillus* and *Gardnerella* are the most abundant microorganisms in the healthy women, while *Atopobium*, *Prevotella*, and *Streptococcus* are most abundant in the RM group (Zhao et al., 2021).

According to the taxonomic analysis, three species of phyla (*Firmicutes*, *Actinobacteria*, and *Bacteroidetes*) were not identical in these two groups. In the RM group, species richness increased and diversity decreased. Due to some research, *Gardnerella vaginalis*, *Prevotella*, *Bacteroides*, and *Veillonella* increase miscarriages and *Sneathia* sanguinegens, in particular, may play an important role in RS (Zhao et al., 2021). Increases in *Atopobium*, *Prevotella*, *Streptococcus*, and *Megasphaera* and decreases in *Lactobacillus* have been noted (Zhang et al., 2019; Zhao et al., 2021). Group B streptococci are a major cause of abortion. Decreases in *Corynebacterium*, *Rhodococcus*, *Sphingomonas*, *Burkholderia*-Caballeronia, *Paraburkholderia*, and *Pseudomonas* are the main bacterial differences between the RS and control groups, indicating their role in miscarriages (Shafiei et al., 2019; Zhao et al., 2021).

Individuals with recurrent miscarriages commonly have vaginal infections such mycoplasma infection, bacterial vaginosis, or fungal vaginosis (Günther et al., 2022). Increased levels of *Proteobacteria*, *Mobiluncus*, *Prevotella*, *Gardnerella vaginalis*, and *Mycoplasma hominis*, as well as *Ureaplasma urealyticum*, *Ureaplasma parvum*, *Firmicutes*, *Atopobium*, *Fusobacteria*, *Sneathia*, *Megasphaera*, and *Streptococcus*, increase the risk of abortion. On the other hand, decreasing *Actinobacteria*, *Bacteroidetes*, *L. crispatus, L.iners, L. gasserii, L. jensenii*, and *Leuconostoc* cause a high risk of abortion. The host’s overall and reproductive physiology are impacted by the vaginal microbiota. There is growing evidence linking vaginal health, pregnancy, and the homeostasis of the vaginal microbiota. Immunological responses and altered microbiota may combine to influence reproductive outcomes (X. Liu et al., 2021).

BV, also known as vaginal bacteriosis or *Gardnerella vaginitis*, is a disease of the vagina caused by excessive bacteria (Oriji et al., 2015). Bacterial vaginosis increased the risk of spontaneous abortion by fivefold (Mansour et al., 2022), especially when *Gardnerella vaginalis* or *Mycoplasmas* were present. A correlation between BV and ART failure, including lower rates of clinical pregnancy and an association with implantation failure, early miscarriage, and preterm birth, has been found (Mansour et al., 2022). BV, sexually transmitted diseases, prematurity (PTB), gynecologic cancer, prenatal fetal membrane rupture, and polycystic ovary syndrome (PCOS) have all been linked to an unbalanced vaginal microbiota (Salek Farrokhi et al., 2019; Zhao et al., 2021). About 40% of infertile patients have chronic endometritis, which can be asymptomatic and may result in recurring implantation failure or even recurrent miscarriage (Elnashar, 2021). On the other side, high estrogen levels can encourage lactobacilli to break down lactic acid and glycogen. By the way, lactobacillus synthesis and bacterial defense benefit from a low vaginal pH (<4.5). Genital infections, which are a major cause of miscarriage, can be prevented in large part thanks to Lactobacillus species. Hence, the pH of the vagina might be a sign of vaginal infections (Guang et al., 2022). While initially not a sign of sickness, this reduces the vagina’s physiological capacity for the development of harmful bacteria, which leads to a rise in vaginal infections (Günther et al., 2022).

An earlier study found that *L.iners* does not support the maintenance of a stable vaginal microbiota during pregnancy but is instead linked to the proliferation of other bacterial genera. The association between *L.iners*, the dominant member of the vaginal microbiota, and a history of spontaneous abortions is consistent with this finding. In first-time mothers, a non-lactobacilli-dominated vaginal microbiota may increase the risk of bacterial migration into the uterus and interfere with the immunological regulatory systems necessary for healthy embryo intrauterine implantation (Nasioudis et al., 2017). Harmful bacteria from the vagina can migrate to the uterus and other parts of the reproductive system. This translocation can cause infections in the endometrium and placenta, leading to complications such as preterm labor and miscarriage (De Siena et al., 2021). Euploid abortion is associated with a significantly higher prevalence of *Lactobacillus* spp. When comparing the reduction of the vaginal microbial population to aneuploid abortion, the immunological profiles of concordant cervicovaginal fluids showed that *Lactobacillus* spp. Decreases in the vaginal microbiota associated with proinflammatory cytokine levels are most pronounced in euploid abortions compared with viable pregnancies (Grewal et al., 2022). A lower abundance or proportion of *Lactobacillus* and a higher abundance of facultative and obligate anaerobes, such as *Gardnerella*, *Prevotella*, *Atopobium*, or *Sneathia*, seem to be associated with a higher risk of contracting sexually transmitted diseases (STDs), such as (HIV), gonorrhea, *Chlamydia*, *Trichomonas*, herpes simplex virus 2 (Günther et al., 2022).

Vaginal to uterine transmission of vaginal microbiota imbalances can result in the activation of chemokines and a local immunological response. Recurrent miscarriages might result from this because it could affect the local immune system’s microcirculation (Günther et al., 2022). Different medications, alterations in the environment, changes in the host’s hormone levels, and immune system can all have an impact on the variety of the microbiota in the female reproductive tract. Several studies have shown that unfavorable pregnancy outcomes are directly related to the microbial balance in the reproductive tract. A cause or an impact of the altered vaginal microbiome composition might be the occurrence of abnormalities in the vaginal microbiome profile in patients with RM and miscarriage (Zhang et al., 2019) (**Figure 3**).

## 6 Conclusion

Differences in the composition of the vaginal microbiome do not have pathogenic consequences in every woman. Several major factors maintain or disrupt the vaginal microbiome including ethnic group, menstruation cycle, and pregnancy which are discussed in this section. The outcome of pregnancy also depends on genetics, age, immune system, and environmental factors. In the index pregnancy, the vaginal microbiota of women who had already given birth, or had just experienced an induced or spontaneous abortion, was qualitatively and quantitatively different from that of women who were having their first child. Early pregnancy vaginal microbiome depletion is a risk factor for early pregnancy miscarriage. Although, early pregnancy miscarriage is not always caused by a high bacterial diversity and quantity of *lactobacilli*. *Lactobacillus* protects against pathogens through the production of antibacterial compounds such as lactic acid and bacteriocins. A change in the microbiota of the vagina is represented by a decrease in *Lactobacillus* and an overgrowth of *Mobiluncus*, *Prevotella*, *Gardnerella vaginalis*, *Mycoplasma hominis* and *Ureaplasma urealyticum*.
